# The Effect of Olive Fruit Fly *Bactrocera oleae* (Rossi) Infestation on Certain Chemical Parameters of Produced Olive Oils

**DOI:** 10.3390/molecules26010095

**Published:** 2020-12-28

**Authors:** Vasilij Valenčič, Bojan Butinar, Maja Podgornik, Milena Bučar-Miklavčič

**Affiliations:** Institute for Oliveculture, Science and Research Centre Koper, Garibaldijeva 1, 6000 Koper, Slovenia; bojan.butinar@zrs-kp.si (B.B.); maja.podgornik@zrs-kp.si (M.P.); milena.bucarmiklavcic@zrs-kp.si (M.B.-M.)

**Keywords:** olive fruit fly, biophenols, sterols, triterpenic dialcohols, fatty acids, “Istrska belica”

## Abstract

Olives affected by active and damaging infestation (olive fruit fly *Bactrocera oleae* (Rossi)) were assayed for their chemical composition. Biophenols were determined by HPLC, sterols, triterpenic dialcohols, and fatty acids by gas chromatography analysis. The acquired data were statistically analyzed. Oils produced from “Istrska belica” fruit affected by active infestation compared to the oils made from fruit affected by damaging infestation showed higher amounts of total oleuropein biofenols (377.3 versus (vs.) 106.6 mg/kg), total biophenols (755 vs. 377 mg/kg), lignans (85.3 vs. 32.9 mg/kg), the dialdehydic form of decarboxymethyl oleuropein aglycone (DMO-Agl-dA) (148.3 vs. 49.0 mg/kg), its oxidized form (DMO-Agl-dA)ox (35.2 vs. 8.5 mg/kg), the dialdehydic form of oleuropein aglycone (O-Agl-dA) (61.1 vs. 8.0 mg/kg), the dialdehydic form of ligstroside aglycone (L-Agl-dA) (63.5 vs. 28.0 mg/kg), the aldehydic form of oleuropein aglycone (O-Agl-A) (40.6 vs. 8.4 mg/kg), and lower amounts of tyrosol (Tyr) (6.0 vs. 13. 9 mg/kg) and the aldehydic form of ligstroside aglycone (L-Agl-A) (13.8 vs. 40.3 mg/kg). Higher values of stigmasterol (2.99%) and lower values of campesterol (2.25%) were determined in oils affected by damaging infestation; an increase in triterpenic dialcohols was also observed (3.04% for damaging and 1.62% for active infestation). Oils affected by damaging infestation, compared to active infestation, showed lower amounts of oleic acid (73.89 vs. 75.15%) and higher amounts of myristic (0.013 vs. 0.011%), linoleic (7.27 vs. 6.48%), and linolenic (0.74 vs. 0.61%) acids.

## 1. Introduction

In 2014, the extremely humid (air humidity: 75%; rainfall: 965 mm) and relatively cool weather conditions (temperatures between 20 and 30 °C) during the vegetation period facilitated the development of the olive fruit fly *Bactrocera oleae* (Rossi) [1,2,3,4,5], which caused serious economic damage to Slovenian olive orchards (75% of the product was lost) [1] and in all the Mediterranean region. 

The olive fruit fly *Bactrocera oleae* (Rossi) causes damage by laying eggs under the skin of the fruit. After hatching, larvae feed on the fruit’s mesocarp, causing mechanical destruction of plant tissues [3]. Furthermore, oviposition provides entry for a secondary infestation of bacteria and fungi that rot the fruit and affect the quality of the olive oil produced [3]. Data collected by Malheiro et al. [6] showed that the olive fruit fly attack influenced the chemical, sensory, and nutritional characteristics of oils. Hydrolytic and oxidative processes lead to a significant increase in oil acidity and peroxide value [7,8,9,10] and can be caused by microorganisms associated with the olive fruit fly attack [11,12], especially molds [11] and fungi [12], isolated from infested olive fruits. The olive fly attack is also associated with an increase in the coefficients of specific extinction in the ultraviolet K_232_ and K_270_ of olive oils [7,9,10] and the presence of negative sensory attributes such as fusty, musty, winey, grubby, and rancid [7,9,13,14]. A decrease in total biophenols was also reported [8,9,14]. Olive (*Olea europaea* L.) biophenols are secondary metabolites that protect fruits and olive oil from oxidation and contribute to the sensory characteristics of the produced extra virgin olive oil. Biophenols can be simple substituted compounds with a low molecular weight, having one or more hydroxyl groups attached to the aromatic ring, or more complex structures attached to monoterpenes. The olive belongs to the Oleaceae family, which is characterized by the presence of secoiridoids [15]. Oleuropein and ligstroside, the most significant oleosides in olive fruits, are esters of elenolic acid with 2-(3,4-dihydroxyphenyl)ethanol (hydroxytyrosol (TyrOH)) and 2-(4-hydroxyphenyl)ethanol (tyrosol (Tyr)), respectively [16]. Oleuropein and ligstroside derivatives give extra virgin olive oil a specific aroma and are responsible for the bitter and pungent positive attributes [17]. Changes in biophenol composition due to the attack of the olive fly were also reported [8,18].

There are relatively few recent publications describing the effect of the olive fruit fly on sterol content and composition and triterpenic dialcohol content. Changes regarding total sterols and β-sitosterol were reported [19], but it seems that there are contrasting opinions about Δ-7-stigmastenol content [20,21]. Sterols are also influenced by the variety of olive and the state of ripening of the fruits [22]. There were no data available about the influence of an olive fruit fly attack on triterpenic dialcohols (erythrodiol and uvaol). The sterol content and composition, and the erythrodiol and uvaol content are important parameters for determining the authenticity of an oil; the limit values of the mentioned parameters are purity characteristics listed in Commission Regulation (EEC) No 2568/91, last amended by Commission Implementing Regulation (EU) 2019/1604 of September 27, 2019 [23].

Olive oils are a source of fatty acids, especially monounsaturated fatty acids that contribute to the oxidative stability of olive oils. Some authors [10,24] have reported that there were no significant changes in fatty acid composition due to the olive fruit fly attack, but another study [25] reported a negative correlation between oleic acid content and the infestation level of the olive fruits. 

The aim of this study was to evaluate the influence of active and damaging olive fruit fly infestation on biophenols, sterols, triterpenic dialcohols, and fatty acids. The fruits with active infestation were characterized by the presence of punctures made by olive fruit flies, whereas the fruits with damaging infestation were characterized by the presence of emergence holes. The results of the investigation provide a thorough understanding of the biochemical changes in olives affected by a severe olive fruit fly attack. The infestation affected the sterols, which are vital legal authenticity parameters necessary for the commercial trade of olive oil.

## 2. Results

### 2.1. Biophenol Content and Composition

In the study, we determined the effect of active and damaging olive fruit fly infestation on the biophenol content and composition. Biophenols were determined in olive oils produced from the olive fruits of the “Istrska belica” variety, collected from 10 locations in Slovenian Istria. The results of the means, the standard error of the mean, and the statistically significant differences between active and damaging infestation for the single parameter are presented in Table 1. 

Statistically significant differences (*t*-test, α < 0.05) between active and damaging infestation were determined in the case of tyrosol (Tyr), vanillic and caffeic acids, vanillin, p-coumaric acid, the dialdehydic form of decarboxymethyl oleuropein aglycone (DMO-Agl-dA), oxidised dialdehydic form of decarboxymethyl oleuropein aglycone ((DMO-Agl-dA)ox), dialdehydic form of oleuropein aglycone (O-Agl-dA), lignans (sum of pinoresinol and 1-acetoxy-pinoresinol), dialdehydic form of ligstroside aglycone (L-Agl-dA), aldehydic form of oleuropein aglycone (O-Agl-A), aldehydic form of ligstroside aglycone (L-Agl-A), total oleuropein biophenols, and total biophenols.

Compared to the oils made from fruits affected by damaging infestation, the oils made from fruits affected by active infestation have higher average amounts of DMO-Agl-dA (148.3 versus (vs.) 49.0 mg/kg), (DMO-Agl-dA)ox (35.2 vs. 8.5 mg/kg), O-Agl-dA (61.1 vs. 8.0 mg/kg), L-Agl-dA (63.5 vs 28.0 mg/kg), O-Agl-A (40.6 vs. 8.4 mg/kg), total oleuropein biophenols (377.3 vs. 106.6 mg/kg), total biophenols (755 vs. 377 mg/kg), lignans (85.3 vs. 32.9 mg/kg), vanillin (4.0 vs. 1.6 mg/kg), and p-coumaric acid (11.0 vs. 4.0 mg/kg). The presence of vanillic and caffeic acids were quantified only in samples with active infestation (1.8 mg/kg), whereas in samples with damaging infestation, vanillic and caffeic acids were not detected. The values of Tyr (6.0 vs. 13. 9 mg/kg) and L-Agl-A (13.8 vs. 40.3 mg/kg) were lower in oils affected by active infestation.

The statistical analysis of the data showed that there were no significant differences between active and damaging infestation for the following parameters: hydroxytyrosol (TyrOH), oxidised dialdehydic form of decarboxymethyl ligstroside aglycone ((DML-Agl-dA)ox), dialdehydic form of decarboxymethyl ligstroside aglycone (DML-Agl-dA), and total ligstroside biophenols. Nevertheless, we observed that the mean values of the mentioned parameters were higher in oils affected by an active infestation.

**Figure 1:**
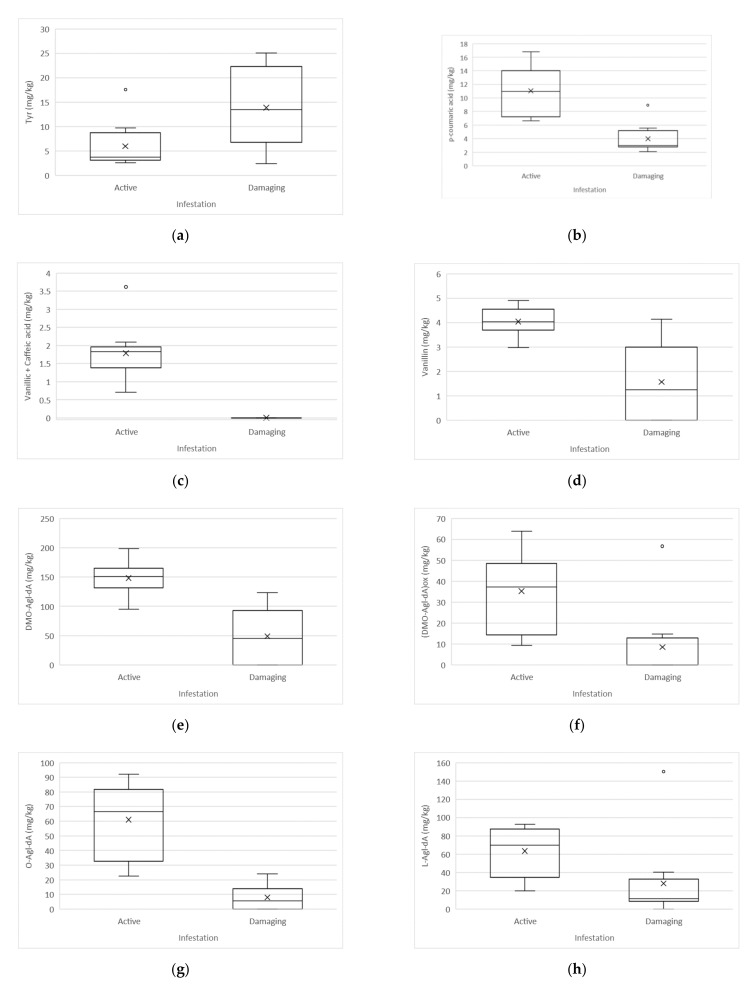
Boxplot representation of statistically significant differences between active and damaging infestation of biophenols (mg/kg): (**a**) Tyr: Tyrosol; (**b**) p-coumaric acid; (**c**) Vanillic acid + Caffeic acid; (**d**) Vanillin; (**e**) DMO-Agl-dA: Decarboxymethyl oleuropein aglycone, dialdehyde form; (**f**) (DMO-Agl-dA)ox: Decarboxymethyl oleuropein aglycone, oxidised dialdehyde form; (**g**) O-Agl-dA: Oleuropein aglycone, dialdehyde form; (**h**) L-Agl-dA: Ligstroside aglycone, dialdehyde form.

**Figure 2:**
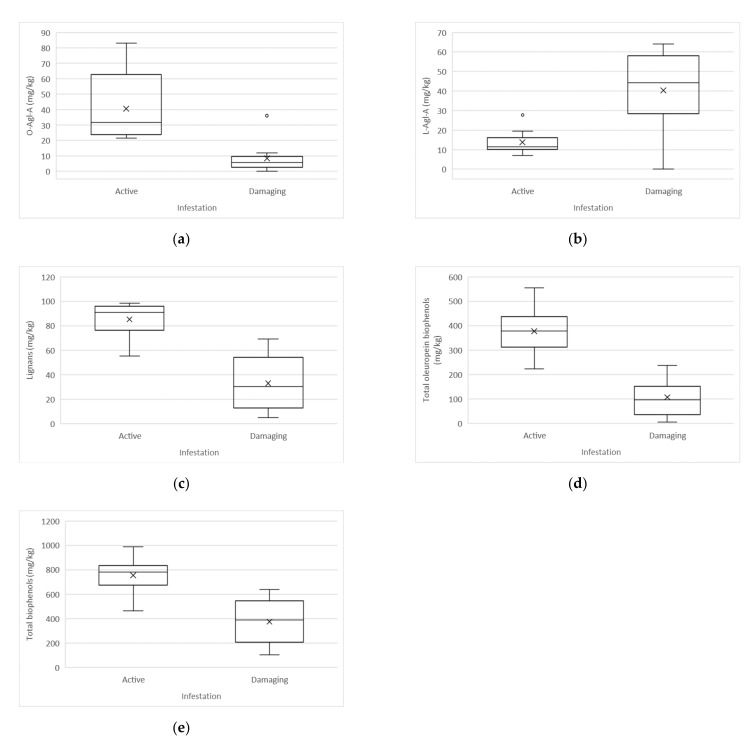
Boxplot representation of statistically significant differences between active and damaging infestation of biophenols (mg/kg): (**a**) O-Agl-A: Oleuropein aglycone, aldehyde form; (**b**) L-Agl-A: Ligstroside aglycone, aldehyde form; (**c**) Lignans: sum of pinoresinol and 1-acetoxy-pinoresinol; (**d**) Total oleuropein biophenols; (**e**) Total biophenols.

Boxplot representation of statistically significant differences between active and damaging infestation of biophenols are shown in Figure 1 and Figure 2.

A paired-sample *t*-test (α < 0.05) was conducted to determine whether there was a correlation between oleuropein and ligstroside derivaties. The following paired-sample *t*-tests were performed: TyrOH and Tyr, DMO-Agl-dA and DML-Agl-dA, (DMO-Agl-dA)ox and (DML-Agl-dA)ox, O-Agl-dA and L-Agl-dA, O-Agl-A and L-Agl-A, total oleuropein biophenols, and total ligstroside biophenols, both in samples with active infection and in samples with damaging infection. The following correlations were statistically significant.

There was a significant difference in the amounts of DMO-Agl-dA (M = 148.3, SD = 28.0) and DML-Agl-dA (M = 82.3, SD = 25.0); t(9) = 7.169, *p* < 0.001 in the samples of oils produced from fruits with active infestation, which showed a higher DMO-Agl-dA content and a lower DML-Agl-dA content. The difference between the two compounds was not significant in oils affected by damaging infestation.

There was a significant difference in the amounts of O-Agl-A (M = 40.6, SD = 22.1) and L-Agl-A (M = 13.8, SD = 5.9); t(9) = 3.461, *p* = 0.007 in the samples of oils produced from fruits with active infestation, which showed a higher O-Agl-A content and a lower L-Agl-A content, whereas, in contrast, oils affected by damaging infestation also showed a significant difference in the amounts of O-Agl-A (M = 8.4, SD = 10.4) and L-Agl-A (M = 40.3, SD = 21.0); t(9) = −4.728, *p* = 0.001, but a higher content of L-Agl-A and a lower content of O-Agl-A were determined.

There was a significant difference in the amounts of total oleuropein biophenols (M= 377.3, SD= 93.8) and total ligstroside biophenols (M = 270.0, SD = 43.3); t(9) = 5.098, *p* = 0.001 in the samples of oils produced from fruits with active infestation, which showed a higher content of total oleuropein biophenols and a lower content of total ligstroside biophenols, whereas, in contrast, oils affected by damaging infestation also showed a significant difference in the amounts of total oleuropein biophenols (M = 106.6, SD = 77.9) and total ligstroside biophenols (M = 223.7, SD = 104.9); t(9) = −4.675, *p* = 0.001, but a higher content of total ligstroside biophenols and a lower content of total oleuropein biophenols were determined.

There was a significant difference in the amounts of TyrOH (M = 2.6, SD = 3.2) and Tyr (M = 13.9, SD = 7.9); t(9) = −5.036, *p* = 0.001 in the samples of oils produced from fruits affected by damaging infestation, which showed a higher Tyr content and a lower TyrOH content. The difference was not significant in oils affected by active infestation.

There were no significant differences between (DMO-Agl-dA)ox and (DML-Agl-dA)ox, and O-Agl-dA and L-Agl-dA, either in oils made from fruits affected by active infestation or in oils affected by damaging infestation. 

### 2.2. Sterol Content and Composition and Triterpenic Dialcohols Content

The sterol content and composition and the content of triterpenic dialcohols erythrodiol and uvaol were determined in oils produced from fruits with active and damaging infestation. The results of the means, the standard error of the mean, and the statistically significant differences between active and damaging infestation for the single parameter are presented in Table 2.

Statistically significant differences (*t*-test, α < 0.05) between active and damaging infestation were determined in the case of stigmasterol, Δ-5,24-stigmastadienol, apparent β-sitosterol, total sterols, erythrodiol, uvaol, and the sum of erythrodiol and uvaol. 

Studying the sterol content and composition, it was seen that the oils produced from fruits affected by active infestation, compared to the oils produced from fruits affected by damaging infestation, have higher average amounts of Δ-5,24-stigmastadienol (1.06 vs. 0.98%) and apparent β-sitosterol (95.65 vs. 93.78%), and lower amounts of stigmasterol (1.20 vs. 2.99%), total sterols (1120 vs. 1437 mg/kg), erythrodiol (1.62 vs. 2.85%), and the sum of erythrodiol and uvaol (1.62 vs. 3.04%). The presence of uvaol was determined only in samples produced from fruits with damaging infestation (0.19%), whereas the presence of uvaol was not detected in samples produced from fruits with active infestation.

The statistical analysis of the data showed that there were no significant differences between active and damaging infestation for the following parameters: cholesterol, 24-methylene-cholesterol, campesterol, campestanol, clerosterol, β-sitosterol, sitostanol, Δ-7-stigmastenol, and Δ-7-avenasterol. Nevertheless, we observed that the average amounts of campesterol, β-sitosterol, sitostanol, and Δ-7-avenasterol were higher in oils produced from fruits affected by damaging infestation.

Brassicasterol, Δ-7-campesterol, and Δ-5,23-stigmastadienol were not detected in oils affected by active infestation nor in oil samples affected by damaging infestation.

**Figure 3:**
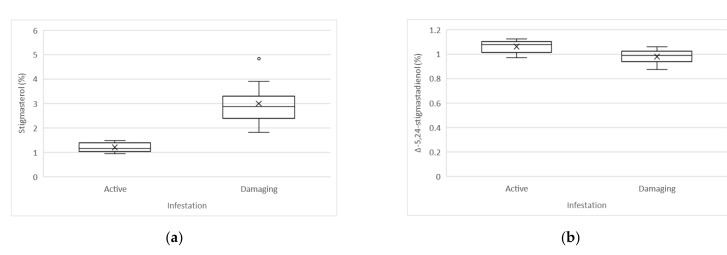

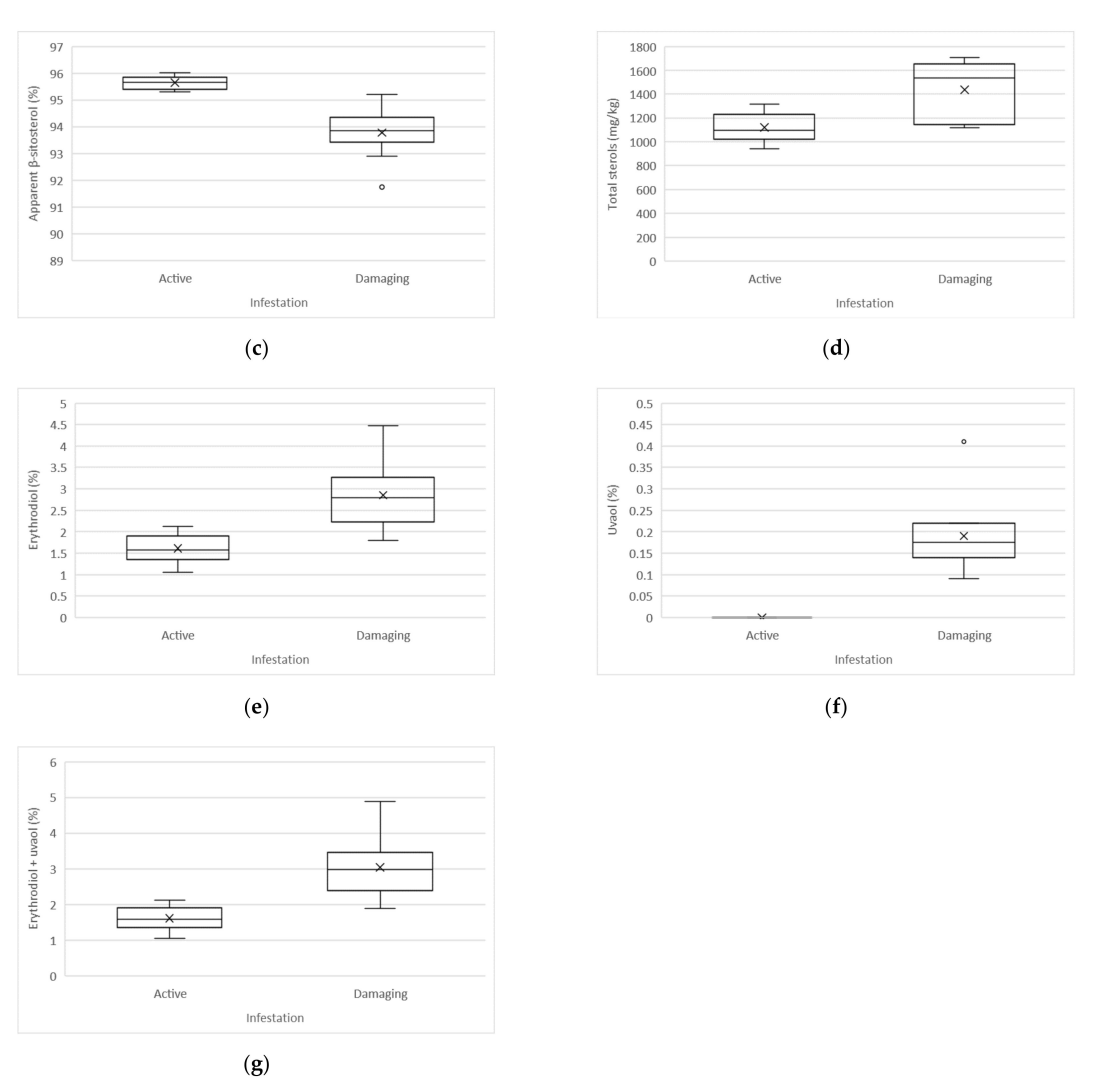
Boxplot representation of statistically significant difference between active and damaging infestation of sterol composition (%), total sterols (mg/kg) and triterpenic dialcohols (%): (**a**) Stigmasterol; (**b**) Δ-5,24-stigmastadienol; (**c**) Apparent β-sitosterol; (**d**) Total sterols; (**e**) Erythrodiol; (**f**) Uvaol; (**g**) Erythrodiol + uvaol.

Boxplot representation of statistically significant differences between active and damaging infestation of sterols and triterpenic dialcohols is shown in Figure 3.

A paired-sample *t*-test (α < 0.05) was conducted to determine whether there was a correlation between stigmasterol and campesterol. There was a significant difference in the amounts of stigmasterol (M = 1.20, SD = 0.19) and campesterol (M = 2.18, SD = 0.23); t(9) = −9.815, *p* < 0.001 in the samples of oils produced from fruits affected by active infestation, which showed a higher campesterol content and a lower stigmasterol content, whereas oils produced from fruits affected by damaging infestation also showed a significant difference in the amounts of stigmasterol (M = 2.99, SD = 0.85) and campesterol (M = 2.25, SD = 0.13); t(9) = 2.995, *p* = 0.015, but a higher stigmasterol content and a lower campesterol content were determined.

### 2.3. Fatty Acid Composition

The fatty acid composition was determined in oils produced from fruits with active and damaging infestation. The results of the means, the standard error of the mean, and the statistically significant differences between active and damaging infestation for the single parameter are presented in Table 3.

Statistically significant differences (*t*-test, α < 0.05) between active and damaging infestation were determined for the content of myristic (C 14:0), oleic (C 18:1), linoleic (C 18:2), and linolenic (C 18:3) acids. Samples of oils produced from fruits affected by active infestation, compared to the oil samples affected by damaging infestation have a higher average amount of oleic acid (75.15 vs. 73.89%) and lower average amounts of myristic (0.011 vs. 0.013%), linoleic (6.48 vs. 7.27%), and linolenic (0.61 vs. 0.74%) acids. 

The statistical analysis of the data showed that there were no significant differences between active and damaging infestation for all other fatty acids reported in Table 3. Nevertheless, we observed that the average amounts of palmitic (C 16:0) and palmitoleic acid (C 16:1) were lower, whereas the stearic acid (C 18:0) was higher in oils produced from fruits affected by active infestation. 

The total saturated fatty acids (SFA), total monounsaturated fatty acids (MUFA), total polyunsaturated fatty acids (PUFA), atherogenic index (AI), and thrombogenic index (TI) were calculated. Statistically significant differences (t-test, α < 0.05) between active and damaging infestation were determined for MUFA, PUFA, and PUFA/SFA ratio. Samples obtained from oils affected by active infestation, compared to the samples taken from oils affected by damaging infestation, have a higher average amount of MUFA (76.93 vs. 75.85%) and lower average amounts of SFA (15.99 vs. 16.13%), PUFA (7.09 vs. 8.01%), and PUFA/SFA ratio (0.44 vs. 0.50). Both AI (0.15) and TI (0.35) values were the same in active and damaging infestation.

**Figure 4:**
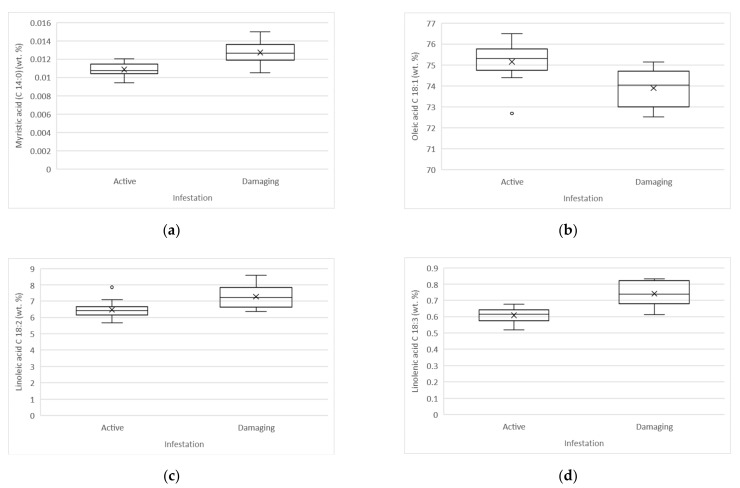
Boxplot representation of statistically significant difference between active and damaging infestation of fatty acid composition (wt. %): (**a**) Myristic acid (C 14:0), (**b**) Oleic acid (C 18:1) (**c**) Linoleic acid (C 18:2), (**d**) Linolenic acid (C 18:3).

Boxplot representation of statistically significant differences between active and damaging infestation of fatty acid composition is shown in Figure 4.

## 3. Discussion

### 3.1. Biophenol Content and Composition

In studying the influence of active and damaging olive fruit fly infestation on the biophenol content and composition, it was found that the oleuropein derivatives DMO-Agl-dA, (DMO-Agl-dA)ox, O-Agl-dA, and O-Agl-A drastically decreased in oils made from fruits affected by damaging infestation. Compared to the active infestation, a statistically significant loss of the mentioned compounds was determined. Observing the ligstroside derivatives, statistically significant differences between the two infestation levels were determined in the cases of L-Agl-dA, L-Agl-A, and Tyr. The amount of L-Agl-dA decreased, whereas the amounts of L-Agl-A and Tyr increased in oils obtained by fruits affected by damaging infestation.

The attack of the olive fruit fly also influenced the simple biophenols where vanillic and caffeic acids were determined only in samples affected by active infestation, whereas in samples affected by damaging infestation, vanillic and caffeic acids were not detected. A significant decrease in p-coumaric acid was determined in oils affected by damaging infestation. The lignans (sum of pinoresinol and 1-acetoxy-pinoresinol) and the total biophenols were also significantly affected by damaging infestation, compared to active infestation, significantly lower amounts of lignans and total biophenols were detected in oils from damaging infestation.

Statistically significant correlations between oleuropein and ligstroside derivaties were determined. Oils made from fruits affected by active infestation showed higher amounts of oleuropein derivaties DMO-Agl-dA, O-Agl-A, and total oleuropein biophenols, and lower amounts of the corresponding ligstroside compounds. Oils affected by damaging infestation showed higher amounts of ligstroside derivaties L-Agl-A, total ligstroside biophenols, and Tyr, and lower amounts of the corresponding oleuropein derivaties. The results suggest that oleuropein derivatives were first affected by the damaging infestation of the olive fruit fly, followed by the ligstroside derivatives, and with the exception of Tyr and L-Agl-A, a general decrease in biophenol compounds was observed due to the damaging infestation.

The statistical data analysis confirmed the preliminary findings of the research previously reported by Matoš [27] regarding the differences between the oil samples affected by active and damaging infestation. Our data are in agreement with Gucci et al. [8], who studied the “Frantoio” variety of olive and stated that the main phenolic compounds influenced by an infestation of the olive fruit fly were the secoiridods, and with Gómez-Caravaca et al. [18], who reported significant losses in simple phenols, lignans, and secoiridoids in several Italian olive cultivars due to an infestation of the olive fruit fly. Data collected by Malheiro et al. [6] showed that the total biophenol amount is negatively correlated with the infestation level of the olives. Koprivnjak et al. [9] reported that the main reasons for the loss of biophenols are most likely an increase in endogenous polyphenoloxidase activity due to the damage of the cellular structure and the exposure to oxygen due to exit holes on the surface of the fruit. Gucci et al. [8], Koprivnjak et al. [9], Mraicha et al. [24], and Tamendjari et al. [25] reported that the loss of total biophenols is also related to the variety of olive. Pavlidi et al. [28] pointed out that *Bactrocera oleae* larvae are one of the few insect larvae capable of feeding on the olive mesocarp and, as reported by Ben-Yosef et al. [29], are able to develop in unripe olive fruits, rich in secondary metabolites. Unripe olives impose a major constraint on developing larvae by reducing the nutritional value of proteins. It was reported that oleuropein is a potent protein cross-linker, which is more active in unripe fruits. Larvae overcome this restriction with symbiotic bacteria, which counteracts the inhibitory effect of oleuropein. Oleuropein aglycones also showed antimicrobial activity, inhibiting the growth of several gram-positive and gram-negative bacteria (Fleming et al. [30]). Ben-Yosef et al. [29] assumed that *Candidatus Erwinia dacicola*, a larvae gut resident often accompanied by a varied consortium of other bacteria, facilitate larval development in unripe olives by securing a source of digestible protein or amino acids for the larvae. The secretion of biophenol-degrading or resistant enzymes may facilitate the dissociation of oleuropein-protein complexes in larval gut and increase dietary protein digestibility. The surviving and developing mechanism of larvae described by Ben-Yosef et al. [29] can be associated with the processes in “Istrska belica”, where a high amount of total biophenols and oleuropein derivatives were determined. Olive oils produced from fruits affected by active infestation had 755 mg/kg total biophenols, whereas their amount decreased to 377 mg/kg in oils obtained from fruits affected by damaging infestation where exit holes were present, which indicates that the reproductive cycle of the larvae had taken place. During feeding, the larva forms tunnels inside the drupe destroying the pulp and facilitating a secondary infestation by bacteria and fungi that causes hydrolytic and oxidative processes [11,12], which can be related to the loss of oleuropein derivatives.

### 3.2. Sterol Content and Composition and Triterpenic Dialcohols Content

The sterol content and composition were also affected by the olive fruit fly infestation. Lower amounts of Δ-5,24-stigmastadienol and apparent β-sitosterol were found in oils produced from fruits affected by damaging infestation, whereas the content of total sterols and stigmasterol significantly increased, compared to the oils produced from fruits affected by active infestation. A correlation between stigmasterol and campesterol related to olive fruit fly infestation was determined. Oils produced from fruits with damaging infestation showed a higher content of stigmasterol and a lower content of campesterol, which is not typical for olive oils and is in contrast with the requirements (stigmasterol < campesterol), set out by Commission Regulation (EEC) No 2568/91, last amended by Commission Implementing Regulation (EU) 2019/1604 of September 27, 2019 [23]. From the legal point of view, such oils cannot be sold on the market. It was also observed that singular samples showed an amount of apparent β-sitosterol, lower than 93.0%, which is the lower limit value prescribed in Commission Regulation (EEC) No 2568/91, last amended by Commission Implementing Regulation (EU) 2019/1604 of September 27, 2019 [23]. 

We found that Δ-7-stigmastenol was not affected by olive fruit fly infestation, and this parameter was consistent with the limit value (≤0.5%), prescribed by Commission Regulation (EEC) No 2568/91, last amended by Commission Implementing Regulation (EU) 2019/1604 of September 27, 2019 [23]. Our data are in agreement with Housheya et al. [21] who reported the olive fruit fly and peacock eye (*Spilocaea oleagina* Cast) disease infection appear to have insignificant effects on the Δ-7-stigmastenol concentration in oils from the “Nabali-Baladi” variety, and with Delrio et al. [19], but are in contrast with Abu-Alruz et al. [20], who reported higher amounts of Δ-7-stigmastenol in the Palestinian olive variety due to the attack of the olive fruit fly, which could be related to a varietal characteristic and/or sensitivity.

Brassicasterol, Δ-7-campesterol, and Δ-5,23-stigmastadienol were not detected either in oils produced from fruits affected by active infestation or in oils produced by fruits affected by damaging infestation, as in Slovenian extra virgin olive oils produced from healthy fruits. Average values for the mentioned sterols and for triterpenic dialcohols in “Istrska belica” oils for the period 2003–2012 are reported by Bandelj et al. [31].The olive fruit fly attack influenced the triterpenic dialcohols. The presence of uvaol was determined only in samples of oil produced from fruits affected by damaging infestation, whereas uvaol was not detected in samples affected by active infestation. The amounts of erythrodiol and the sum of erythrodiol and uvaol were also higher in the case of damaging infestation. It was observed that even in the case of triterpenic dialcohols, singular samples showed very high amounts of the sum of erythrodiol and uvaol, which exceeded the limit value (≤4.5%), set out by Commission Regulation (EEC) No 2568/91, last amended by Commission Implementing Regulation (EU) 2019/1604 of September 27, 2019 [23].

### 3.3. Fatty Acid Composition

Myristic, oleic, linoleic and linolenic acids were affected by the damaging olive fruit fly infestation. A statistically significant decrease in oleic acid was determined, whereas higher values of myristic, linoleic and linolenic acids were found in samples affected by damaging infestation, although generally, small amounts of myristic acid were determined.

The results of the investigation showed that the olive fruit fly did not have any influence on trans-fatty acids. Our findings for oleic acid are in agreement with Mraicha et al. [24] and Tamendjari et al. [25]; in particular, the “Azzeradj” variety was more sensitive to attack by the olive fruit fly, but are in contrast with Pereira et al. [10], who reported that for the “Madural” and ”Verdeal Transmontana” varieties, there were no significant changes in fatty acid composition due to the olive fruit fly attack. Malheiro et al. [6] reported that *Bacrocera oleae* causes quality degradation, namely hydrolysis and oxidation, which causes significant changes to fatty acid composition. It was pointed out that the oxidation of unsaturated fatty acids is positively correlated to the infestation level (Malheiro et al. [6]). As previously mentioned, in the case of the “Istrska belica” variety, a decrease in oleic acid was observed.

Generally, the fatty acid composition of the studied “Istrska belica” variety complied with the requirements of Commission Regulation (EEC) No 2568/91, last amended by Commission Implementing Regulation (EU) 2019/1604 of 27 September 2019 [23]. The fatty acid composition of the oil samples attacked by the olive fruit fly are comparable to the data of “Istrska belica” oils, collected for the period 2003–2012, made from healthy, undamaged, and manually picked drupes in a state of optimal ripeness, as reported by Bandelj et at. [31].

The major MUFA in oils produced from fruits affected by active and damaging infestation was oleic acid (C 18:1), followed by palmitoleic acid (C 16:1), eicosenoic acid (C 20:1), and heptadecenoic acid (C 17:1). The representatives of PUFA were linoleic acid (C 18:2) and linolenic acid (C 18:3); the major SFA representatives were palmitic acid (C 16:0) and stearic acid (C 18:0). The ratios between PUFA and SFA (0.44 for active and 0.50 for damaging infestation) were comparable to Kostik et al. [32] (0.49), whereas Hashempour-Baltork et al. [33] reported higher values (0.66) for olive oils. The Report on Health and Social Subjects [34] recommended the PUFA/SFA ratio of approximately 0.45, which was reached by the studied olive oil samples.

AI (0.15) and TI (0.35) in both active and damaging infestation were in accordance with Hashempour-Baltork et al. [33] (AI = 0.15; TI = 0.38) and lower than 1. Sánchez-Rodríguez et al. [35] reported slightly higher values (AI = 0.303–0.326; TI = 0.513–0.520) for the “Arbequina” variety. AI and TI can be used as predictors or risk factors for cardiovascular diseases and should be kept at low levels in a healthy daily diet (Hashempour-Baltork et al. [33]).

## 4. Materials and Methods 

### 4.1. Olive Material and Olive Oil Production

**Figure 5:**
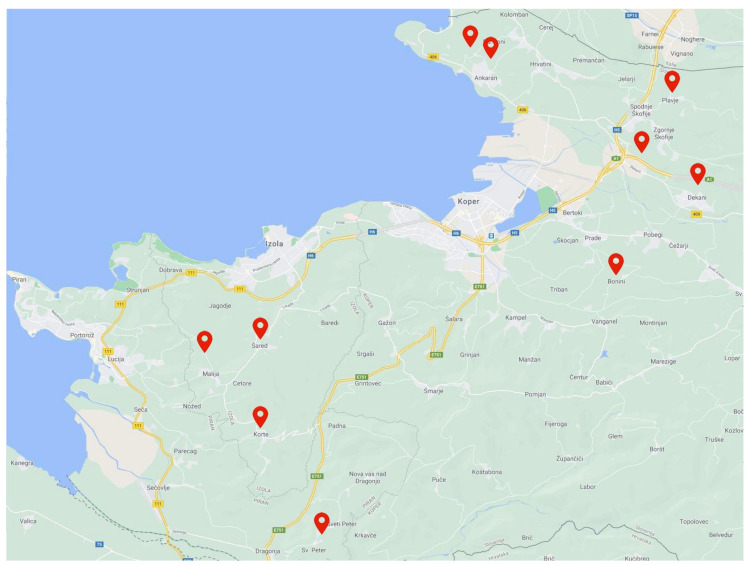
Map showing the 10 sampling locations in Slovenian Istria.

Samples of olives of the “Istrska belica” variety with active and damaging infestation were collected from ten different locations in Slovenian Istria in the period from October 9, 2014, to October 10, 2014. The map of the locations where the samples were taken is shown in Figure 5. The samples from each location were examined and classified into two subsamples according to Petacchi [36]: the fruits with active infestation were characterized by the presence of olive fruit fly punctures, and the fruits with damaging infestation were characterized by the presence of emergence holes. A total of 20 samples were collected, 10 with active infestation and 10 with damaging infestation. For each sample, approximately 1 kg of olive fruits were manually collected and examined. Olive oils were produced in a laboratory olive mill Abencor system MC2 (MC2 Ingenieria y Sistemas, Sevilla, Spain).

### 4.2. Methods

All the methods used for the analysis of biophenols, sterols, triterpenic dialcohols, and fatty acids were accredited in accordance with ISO 17025. All the chemicals reported in the following subsections met the requirements of the official methods and were purchased from Sigma-Aldrich Chemie GmbH (Munich, Germany).

#### 4.2.1. Determination of Biophenols

Biophenol content and composition was determined in accordance with the method accepted by the International Olive Council (IOC), COI/T.20/Doc. No 29 [37]. The extraction was done with a modification of the method from 5 g of oil with the addition of 2.5 mL of internal standard solution (syringic acid 0.15 mg/mL). The sample was transferred to a separatory funnel with the aid of 25 mL of hexane, and the biophenols were extracted three times with methanol/water 60/40 (m/m). The solvent was evaporated with a rotary evaporator at 40 °C and the dry extract was dissolved in 1 mL of methanol. Biophenol content and composition was determined by HPLC analysis as set out in the IOC method. An Agilent 1100 Series HPLC System (Agilent Technologies, Santa Clara, CA, USA) equipped with a binary pump and automatic liquid sampler, C18 reversed-phase column (Phenomenex synergi hydro, 250 × 4.6 mm, 4 µm; Phenomemex, Inc, Torrance, CA, USA), operating at 20 °C, with DAD detection at 280 nm was used. Spectral data for the peaks were recorded in the range of 200–600 nm. The mobile phase used was a gradient consisting of 0.2% aqueous H_3_PO_4_ (by volume) (A) and methanol/acetonitrile 1/1 (by volume) (B). The initial gradient composition was A at 96% and B at 4%. After forty minutes, the ratio of B increased to 50%, to 60% in the next five minutes, and to 100% in the last fifteen minutes. At 72 min from the start, the concentration of B was put at an initial value of 4%. The column was then equilibrated for 10 min before the next injection. A volume of 10 μL of the methanolic extract was injected into the system; the flow rate was 1 mL/min. An external calibration solution of tyrosol (0.030 mg/mL) and syringic acid (0.015 mg/mL) was prepared. All biophenol compounds were quantified using the response factor for tyrosol and assigned by comparing their relative retention times to the retention time of the internal standard syringic acid.

#### 4.2.2. Determination of Sterols and Triterpenic Dialcohols

Sterol content and composition and triterpenic dialcohols content were determined in accordance with Commission Regulation (EEC) No 2568/91, Annex V [38]. The sample preparation involved the addition of the internal standard solution of α-cholestanol (0.2%, m/V), saponification with 2-M ethanolic potassium hydroxide solution, solvent extraction of unsaponifiable matter with diethyl ether, separation of sterol and triterpenic dialcohols from the unsaponifiable matter with thin-layer chromatography, derivatisation into trimethylsilyl ethers, and determination by gas chromatography. An Agilent HP 6890 Series (Agilent Technologies, Santa Clara, CA, USA), equipped with Supelco SPB-5 Capillary GC Column (60 m × 0.53 mm ID, df 5.00 μm; Supelco Inc, Bellefonte, PA, USA) and FID detector was used. The sterols and triterpenic dialcohols were assigned by comparing their relative retention times to the retention time of the internal standard α-cholestanol.

#### 4.2.3. Determination of Fatty Acids

Fatty acid composition was determined in accordance with Commission Regulation (EEC) No 2568/91, Annex Xa and Annex Xb [39]. Fatty acid methyl esters were prepared in heptane with 2-M methanolic potassium hydroxide solution and determined by gas chromatography. An Agilent HP 6890 Series (Agilent Technologies, Santa Clara, CA, USA), equipped with Supelco 2560 Capillary GC Column (100 m × 0.25 mm ID, df 0.20 μm; Supelco Inc., Bellefonte, PA, USA) and FID detector was used. Fatty acids were assigned by comparing the retention times with those of the reference standard Supelco 37 Component FAME Mix.

#### 4.2.4. Statistical Analysis

All the data were statistically analyzed and expressed as mean values ± standard error of the mean (SEM). Significance of the difference was analyzed by ANOVA, *t*-test for equality of means, and paired-samples *t*-test for correlations were performed with SPSS Statistics v. 26 (SPSS, Chicago, IL, USA), with statistical significance set at *p* < 0.05.

## 5. Conclusions

The results of this investigation highlighted that the oleuropein derivatives DMO-Agl-dA (148.3 mg/kg), (DMO-Agl-dA)ox (32.5 mg/kg), O-Agl-dA (61.1 mg/kg), O-Agl-A (40.6 mg/kg), and total oleuropein biophenols (377.3 mg/kg) were predominant in oils produced from fruits affected by active infestation, whereas ligstroside derivatives L-Agl-A (40.3 mg/kg) and Tyr (13.9 mg/kg) were more present in oils produced from fruits affected by damaging infestation, which also affected the absolute loss of total biophenols (755 mg/kg in oils produced from fruits affected by active infestation and 377 mg/kg in oils affected by damaging infestation). Higher values of vanillic and caffeic acids (1.8 mg/kg), vanillin (4.0 mg/kg), and p-coumaric acid (11.0 mg/kg) were determined in oils produced from fruits affected by active infestation than in oils affected by damaging infestation. Biophenol content and composition were more preserved in oils made from fruits affected by active infestation. Oils produced from fruits affected by damaging infestation showed a higher content of stigmasterol (2.99%) and a lower content of campesterol (2.25%), which is not in accordance with the limit values of Commission Regulation (EEC) No 2568/91, last amended by Commission Implementing Regulation (EU) 2019/1604 of 27 September 2019 [23]. Compared to active infestation, oils from fruits affected by damaging infestation had higher amounts of erythrodiol and uvaol (3.04%), myristic (0.013%), linoleic (7.27%), and linolenic (0.74%) acids and a lower amount of oleic acid (73.89%). 

The results of the research highlighted that the relationships between oleuropein and ligstroside derivatives, and stigmasterol and campesterol can be useful markers for distinguishing between oils produced from fruits affected by active and damaging olive fruit fly infestation. 

## Figures and Tables

**Table 1:** Biophenol content and composition of olive oils produced from olive fruits with active infestation and damaging infestation, and *p* values.

Parameter	Active Infestation(mg/kg)	Damaging Infestation(mg/kg)	*p*-Value
TyrOH	6.6 ± 2.6	2.6 ± 1.0	0.174
Tyr	6.0 ± 1.5 *	13.9 ± 2.5 *	0.014
Vanillic acid + Caffeic acid	1.8 ± 0.3 *	N.D. *	<0.001
Vanillin	4.0 ± 0.2 *	1.6 ± 0.5 *	<0.001
p-coumaric acid	11.0 ± 1.1 *	4.0 ± 0.7 *	<0.001
DMO-Agl-dA	148.3 ± 8.9 *	49.0 ± 15.8 *	<0.001
(DMO-Agl-dA)ox	35.2 ± 5.8 *	8.5 ± 5.6 *	0.004
O-Agl-dA	61.1 ± 7.9 *	8.0 ± 2.7 *	<0.001
(DML-Agl-dA)ox	32.8 ± 7.5	21.4 ± 16.0	0.527
DML-Agl-dA	82.3 ± 7.9	63.3 ± 18.2	0.350
Lignans	85.3 ± 4.5 *	32.9 ± 7.0 *	<0.001
L-Agl-dA	63.5 ± 8.7 *	28.0 ± 14.1 *	0.046
O-Agl-A	40.6 ± 7.0 *	8.4 ± 3.3 *	0.001
L-Agl-A	13.8 ± 1.9 *	40.3 ± 6.6 *	0.001
Total oleuropein biophenols	377.3 ± 29.7 *	106.6 ± 24.6 *	<0.001
Total ligstroside biophenols	270.0 ± 13.7	223.7 ± 33.2	0.214
Total biophenols	755 ± 45 *	377 ± 56 *	<0.001

Abbreviations: TyrOH: Hydroxytyrosol; Tyr: Tyrosol; DMO-Agl-dA: Decarboxymethyl oleuropein aglycone, dialdehyde form; (DMO-Agl-dA)ox: Decarboxymethyl oleuropein aglycone, oxidised dialdehyde form; O-Agl-dA: Oleuropein aglycone, dialdehyde form; (DML-Agl-dA)ox: Decarboxymethyl ligstroside aglycone, oxidised dialde-hyde form; DML-Agl-dA: Decarboxymethyl ligstroside aglycone, dialdehyde form; lignans: sum of pinoresinol and 1-acetoxy-pinoresinol; L-Agl-dA: Ligstroside aglycone, dialdehyde form; O-Agl-A: Oleuropein aglycone, aldehyde form; L-Agl-A: Ligstroside aglycone, aldehyde form; N.D.: not detected. Data are expressed as means ± standard error of the mean (SEM), *n* = 10, * *p* < 0.05: statistically significant differences between active and damaging infestation for the single parameter.

**Table 2:** Sterol content and composition and erythrodiol and uvaol content of olive oils produced from olive fruits with active infestation and damaging infestation, *p* values and limit values for extra virgin olive oils in accordance with Commission Regulation (EEC) No 2568/91, last amended by Commission Implementing Regulation (EU) 2019/1604 [23].

Parameter	Unit	Active Infestation	Damaging Infestation	*p*-Value	Limit Value
Cholesterol	%	0.10 ± 0.01	0.09 ± 0.01	0.589	≤0.5
24-methylene-cholesterol	%	0.27 ± 0.01	0.24 ± 0.01	0.198	-
Campesterol	%	2.18 ± 0.07	2.25 ± 0.04	0.407	≤4.0
Campestanol	%	0.09 ± 0.009	0.09 ± 0.005	0.689	-
Stigmasterol	%	1.20 ± 0.06 *	2.99 ± 0.27 *	< 0.001	<Campesterol
Clerosterol	%	0.96 ± 0.03	0.95 ± 0.03	0.899	-
β-sitosterol	%	68.80 ± 1.28	69.03 ± 0.95	0.888	-
Sitostanol	%	1.39 ± 0.10	1.55 ± 0.08	0.243	-
Δ-5-avenasterol	%	23.43 ± 1.43	21.27 ± 0.94	0.222	-
Δ-5,24-stigmastadienol	%	1.06 ± 0.02 *	0.98 ± 0.02 *	0.003	-
Δ-7-stigmastenol	%	0.09 ± 0.005	0.10 ± 0.005	0.739	≤0.5
Δ-7-avenasterol	%	0.42 ± 0.03	0.46 ± 0.03	0.358	-
Apparent β-sitosterol	%	95.65 ± 0.08 *	93.78 ± 0.30 *	< 0.001	≥93.0
Total sterols	mg/kg	1120 ± 38 *	1437 ± 76 *	0.002	≥1000
Erythrodiol ^1^	%	1.62 ± 0.11 *	2.85 ± 0.26 *	< 0.001	-
Uvaol ^1^	%	N.D. *	0.19 ± 0.03 *	< 0.001	-
Erythrodiol ^1^ + uvaol ^1^	%	1.62 ± 0.11 *	3.04 ± 0.28 *	< 0.001	≤4.5

Brassicasterol, Δ-7-campesterol, and Δ-5,23-stigmastadienol were not detected in any samples. The limit value of brassicasterol for extra virgin olive oils is ≤ 0.1% (Commission Regulation (EEC) No 2568/91, last amended by Commission Implementing Regulation (EU) 2019/1604) [23]. ^1^ Erythrodiol and uvaol are expressed as a percentage of total sterols. Abbreviation N.D.: not detected. Data are expressed as means ± standard error of the mean (SEM), *n* = 10, * *p* < 0.05: statistically significant differences between active and damaging infestation for the single parameter. Limit value: limit values for extra virgin olive oils in accordance with Commission Regulation (EEC) No 2568/91, last amended by Commission Implementing Regulation (EU) 2019/1604 [23].

**Table 3:** Fatty acid composition of olive oils produced from olive fruits with active infestation and damaging infestation, *p* values and limit values for extra virgin olive oils in accordance with Commission Regulation (EEC) No 2568/91, last amended by Commission Implementing Regulation (EU) 2019/1604 [23].

Parameter	Unit	Active Infestation	Damaging Infestation	*p*-Value	Limit Value
Myristic acid (C 14:0)	%	0.011 ± 0.0002 *	0.013 ± 0.0004 *	0.001	≤0.03
Palmitic acid (C 16:0)	%	12.35 ± 0.17	12.57 ± 0.07	0.248	7.50–20.00
Palmitoleic acid (C 16:1)	%	1.35 ± 0.08	1.53 ± 0.05	0.065	0.30–3.50
Heptadecanoic acid (C 17:0)	%	0.05 ± 0.002	0.05 ± 0.002	0.562	≤0.40
Heptadecenoic acid (C 17:1)	%	0.10 ± 0.002	0.10 ± 0.003	0.077	≤0.60
Stearic acid (C 18:0)	%	2.83 ± 0.09	2.74 ± 0.06	0.425	0.50–5.00
Oleic acid (C 18:1)	%	75.15 ± 0.34 *	73.89 ± 0.30 *	0.013	55.00–83.00
Linoleic acid (C 18:2)	%	6.48 ± 0.20 *	7.27 ± 0.24 *	0.019	2.50–21.00
Linolenic acid (C 18:3)	%	0.61 ± 0.02 *	0.74 ± 0.02 *	< 0.001	≤1.00
Arachidic acid (C 20:0)	%	0.50 ± 0.01	0.50 ± 0.01	0.917	≤0.60
Eicosenoic acid (C 20:1)	%	0.32 ± 0.005	0.33 ± 0.004	0.300	≤0.50
Behenic acid (C 22:0)	%	0.15 ± 0.005	0.16 ± 0.004	0.189	≤0.20
Lignoceric acid (C 24:0)	%	0.09 ± 0.003	0.09 ± 0.002	0.616	≤0.20
*trans*-oleic isomer (C 18:1 T)	%	0.019 ± 0.001	0.018 ± 0.001	0.235	≤0.05
*trans*-linoleic isomer (C 18:2 CT)	%	0.011 ± 0.001	0.012 ± 0.001	0.075	-
*trans*-linolenic isomer (C 18:3 CTC)	%	0.013 ± 0.001	0.011 ± 0.001	0.226	-
Total *trans*-linoleic and *trans*-linolenic isomers (C 18:2 CT + C 18:3 CTC)	%	0.023 ± 0.001	0.024 ± 0.001	0.856	≤0.05
SFA	%	15.99 ± 0,10	16.13 ± 0,06	0.228	-
MUFA	%	76.93 ± 0,28 *	75.85 ± 0,29 *	0.016	-
PUFA	%	7.09 ± 0,19 *	8.01 ± 0,26 *	0.010	-
PUFA/SFA ratio	-	0.44 ± 0,01 *	0.50 ± 0,02 *	0.008	-
AI	-	0.15 ± 0,002	0.15 ± 0,001	0.222	-
TI	-	0.35 ± 0,003	0.35 ± 0,002	0.808	-

Abbreviations: SFA: saturated fatty acids; MUFA: monounsaturated fatty acids, PUFA: polyunsaturated fatty acids; AI: atherogenic index, AI = (4 × C14:0 + C16:0)/(MUFA + PUFA); TI: thrombogenic index, TI = (C14:0 + C16:0 + C18:0)/[(0.5 ×MUFA) + (0.5 × PUFA_(n-6)_) + (3 × PUFA_(n-3)_) + (PUFA_(n-3)_/PUFA_(n-6)_)]. AI and TI were calculated in accordance with Ulbricht and Southgate, 1991 [26]. Data are expressed as means ± standard error of the mean (SEM), *n* = 10, * *p* < 0.05: statistically significant differences between active and damaging infestation for the single parameter. Limit value: limit values for extra virgin olive oils in accordance with Commission Regulation (EEC) No 2568/91, last amended by Commission Implementing Regulation (EU) 2019/1604 [23].

## Data Availability

Not applicable.
